# Temporal and spatial dynamics of amphioxus population (*Branchiostoma belcheri tsingtaneuse*) and its influential factors in Luan River Estuary, China

**DOI:** 10.1002/ece3.1152

**Published:** 2014-07-07

**Authors:** Luo Hao, Ma Minghui, Liang Bin, Bao Chenguang

**Affiliations:** Department of Strategy Research and Information Services, National Marine Environmental Monitoring Center, State Oceanic AdministrationLiaoning, China

**Keywords:** Biomass, *Branchiostoma belcheri tsingtauense*, grain size, population density, population dynamics

## Abstract

Population distribution of amphioxus (*Branchiostoma belcheri tsingtauense*) was investigated in the Luan River Estuary from 1999 to 2011, to describe its trends and discover the factors that influence its decline. Amphioxus are distributed on the seabed at 5–10 m depth, between the Xinkai Estuary and the Dapu River Estuary, where the primary sand components of the sediment are medium and fine, with particle size ranging from 0.001 to 1 mm. In recent years, the population density and biomass of amphioxus have sharply decreased. Changes in sediment granularity composition may significantly influence amphioxus distribution. Our data showed that the highest density region of amphioxus occurred where >90% of the sediment particles were between 0.063 and 0.5 mm in size. A reduction in sediment discharge from Luan River and the expansion of raft-breeding mariculture may be causing the decline in amphioxus through habitat destruction.

## Introduction

Amphioxus (*Branchiostoma belcheri tsingtauense*), a cephalochordate, is regarded as a living fossil. Amphioxus is the closest extant relative of the vertebrates and occupies an extremely important phylogenetic position in the evolution of vertebrates. Amphioxus is primarily distributed in tropical and subtropical shallow seas, where water depths range from 8 to 16 m and between 40° north and 48° south. Three species are distributed in the shallows of the Bohai Sea, Yellow Sea, East China Sea and South China Sea, in the genera Branchiostoma, Epigonichthys, and Amphioxus. (Xiaomei and Shaohua 1997; Luna et al. 2005; Yiquan and Shaohua 2005) *B. belcheri tsingtauense*, the species examined in the present study, is a subspecies of *B. belcheri*, and the Luan River Estuary is its only high-density habitat in Bohai Bay (Gao et al. 2000; Qian et al. 2003).

In recent years, following the rapid development of the inshore economy, environmental pressures on the inshore sea has increased (Bulletin of China's Marine Environmental Status of China [Bibr b1]). Amphioxus populations are threatened by discharge of land-sourced pollutants into the sea, sand mining and sediment transportation, illegal fishing (Guoshou et al. 1996), aquaculture expansion, and increasing maritime traffic (Jingrong, et al. 1995) which both kills amphioxus and destroys their habitat (Shaosai et al. 2003). No recent studies have examined the long-term population dynamics of amphioxus species, so it is difficult to answer what led to population dynamics. In this study, we investigated the status and dynamic trends of the amphioxus population in the Luan River Estuary from 1999 to 2011 in order to identify the primary environmental impacts on amphioxus and provide a scientific basis for a conservation program.

## Materials and Methods

### Study area and site

**Figure 1:**
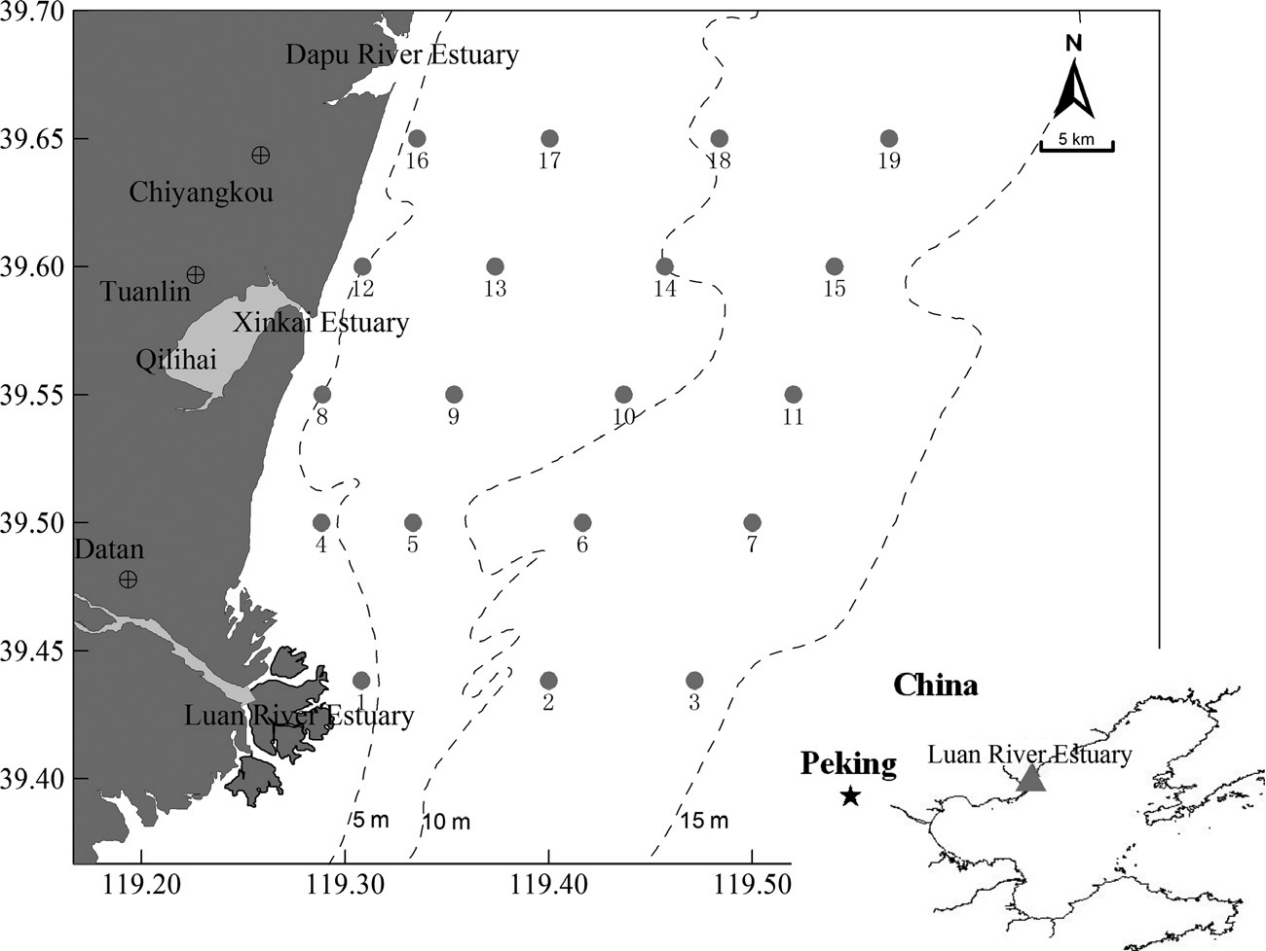
Location of sampling stations in the Luan River Estuary, China (Map of the study area in the Luan River Estuary, China, showing the sample sites. Solid circles represent stations, and dotted lines show depth gradients).

The study area extended from the high-tide line to the 15 m depth contour offshore, between 39°25′ and 39°39′ latitude, including approximately 300 km^2^ of the Luan River Estuary, China (Fig. 1). We used a network of 23 sampling stations in the area.

### Environmental data survey

The physical and chemical parameters of seawater were measured at each station by multiparameter water probe meters (Quanta G, Hydro lab Co., Ltd).

### Sample collection

To determine the distribution of amphioxus within the habitat, we collected samples by digging with a Petersen grab (0.05 m^2^) to a depth of 0–10 cm below the sediment surface each August, from 1999 to 2011. We typically dredged four times in each area sampled, and each area was 0.2 m^2^. Tidal currents flowing at a maximum speed of two knots prevented us from dredging at precisely the same location each time. Sediment samples including amphioxus were sieved through a sieve with 0.5 mm mesh. Specimens were fixed and preserved in 4% buffered formalin.

We also collected 5–10 L of sand from each sampling site, which was then taken to the laboratory for particle size analysis. Each sample of ca. 500 g of sand was passed through a series of sieves (mesh size: 1.0, 0.5, 0.25, 0.125, and 0.063 mm), and the different-sized fractions were weighed after being dried at 80°C for 3 days. The percentage of each particle size, relative to the dry weight of the entire sample, was calculated.

### Data analysis

1. The number of amphioxus individuals was counted in the laboratory, and their biomass (wet weight) was weighed on an electronic scale (precision 0.01 g).
2. The species distribution along the sea transects was displayed using GIS.
3. Particle size distribution contours were extrapolated using the particle data obtained for each sampling point using the Kriging methodology, following the method described by Golden Software Surfer 8 to determine the sediments from predicted values.
4. Correlations between environmental factors and amphioxus density and biomass were calculated using SPSS 13.0: SPSS Inc. Chicago, Illinois.
5. Sand percentages of different granularity were calculated and compared with average biomass and density. To analyze correlations between amphioxus distribution and sediment using hierarchical clustering, data were parameterized by sum standardization, considering the data's proximity measure in Euclidean distance and using the nearest neighbor method to form clusters. All statistical analyses were conducted using SPSS 13.0.

## Results

### Environmental data

**Figure 2:**
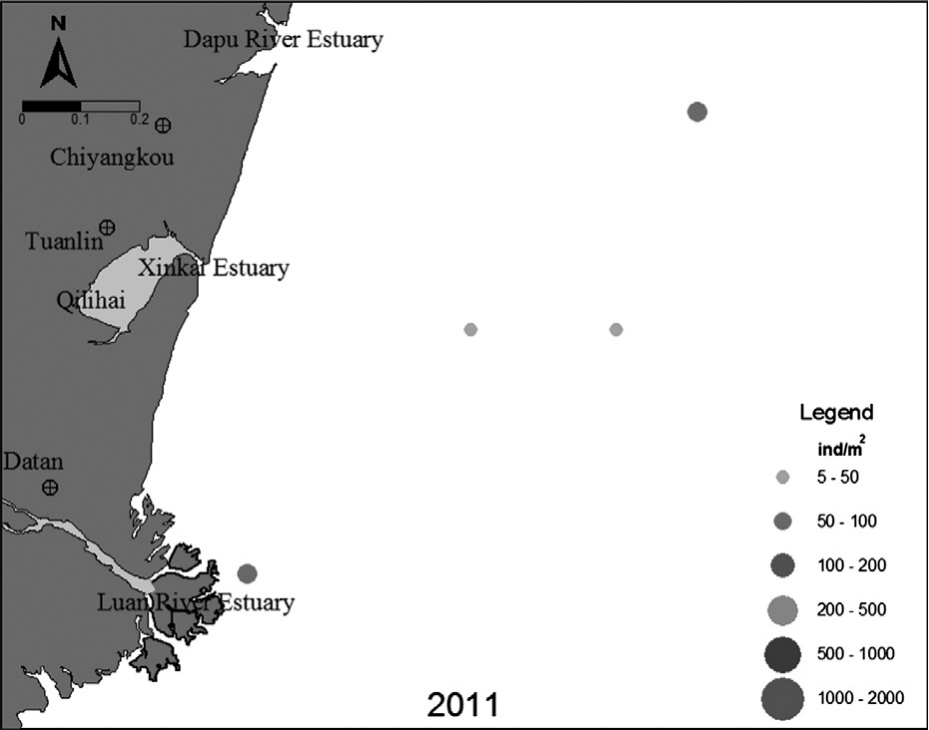
Spatial distribution patterns of the amphioxus population in August 2011 (Solid circles represent the amphioxus population densities, and circles with different diameters represent different population densities).

The sampling stations comprised a depth gradient that varied from 5.0 to approximately 14.0 m deep (Fig. 2). In May and August 2011, seawater temperatures displayed seasonal variation typical for temperate regions, with higher temperatures in August (15.27°C) and lower temperatures in May (25.88°C). Salinity (31.7 ± 0.7) and pH (8.13 ± 0.4) showed no significant variation over space or time. Dissolved oxygen varied between May (10.73 mg/L) and August (9.15 mg/L).

### Spatial distribution of population

*Branchiostoma belcheri tsingtauense* inhabit the sandy seafloor between 5 and 15 m depth. The annual mean density of amphioxus in summer (August 2011) was 53 ind/m^2^. The greatest density (70 ind/m^2^) was observed at sample station l, near the low-water mark; the lowest density (40 ind/m^2^) was observed at station 10, at a depth of between 10 and 15 m and far from the low-water mark (Fig. 2).

**Figure 3:**
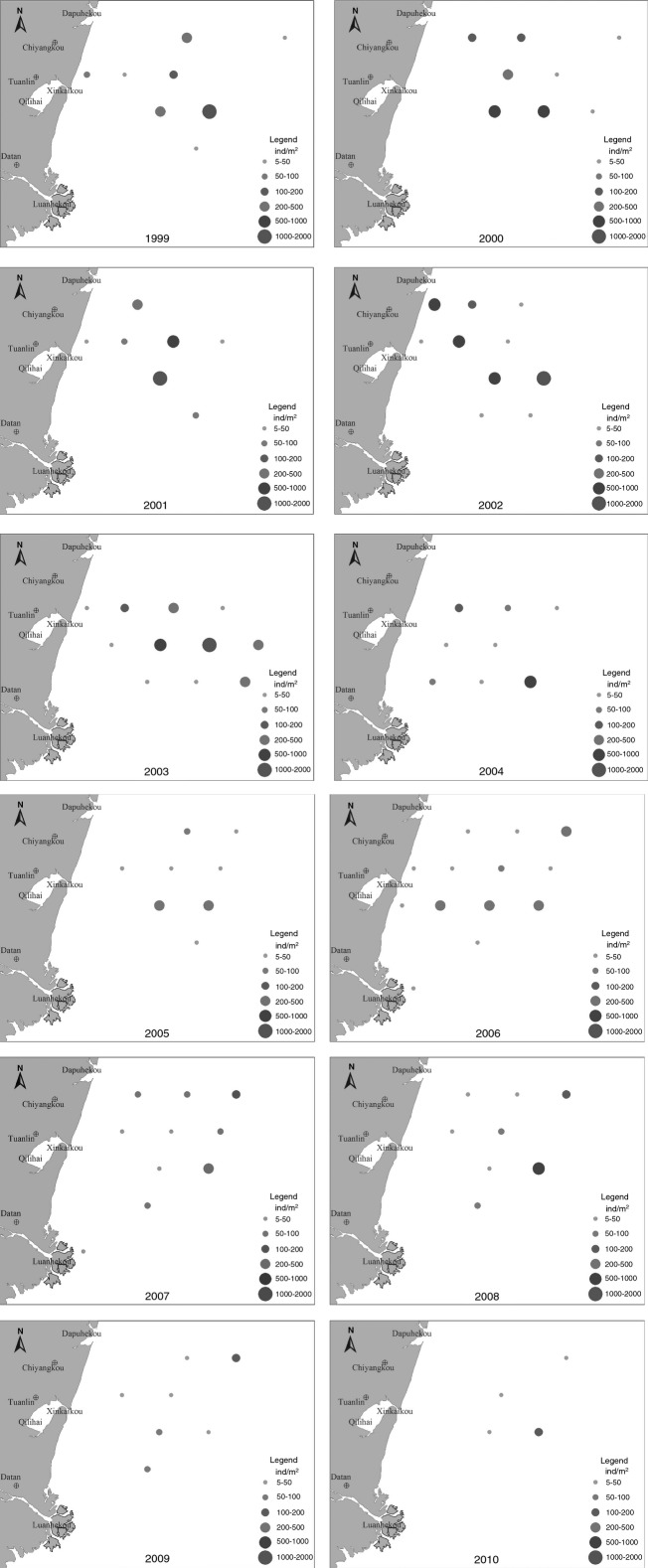
Temporal and spatial dynamics of the amphioxus population between 1999 and 2010 (Solid circles represent amphioxus density and circles with different diameters represent different population densities).

Between 1999 and 2003, areas of distribution of *B. belcheri tsingtauense* were clustered offshore, between the Dapu River Estuary and the Xinkai Estuary, at a depth of 0–15 m. The greatest density was observed at stations 10 and 11. From 2004 to 2008, amphioxus were found everywhere between the Dapu River Estuary and the Luan River Estuary, but the core distribution zone shifted to outside the Xinkai Estuary, at a depth of 5–15 m. After 2009, amphioxus could not be found near shore, only in deep water. Amphioxus was distributed near the shores of the Luan River Estuary in 2008, 2010, and 2011, but the primary distribution was located between the Dapu River Estuary and the Xinkai Estuary, at a depth of 5–15 m (Fig. 3).

### Densities and biomass

The density and biomass of amphioxus changed annually. From 1999 to 2011, the general trend of biomass and density was descending; from 1999 to 2001, the trend was upward, and the greatest abundance (515 ind/m^2^ and 17.7 g/m^2^) was recorded in August 2001. Subsequently, the trend was downward, and the lowest abundance (29 ind/m^2^ and 2.64 g/m^2^) was recorded in 2010.

### Relationship between environment and amphioxus distribution

#### Relationship between aquatic environmental factors and amphioxus distribution

Correlations between environmental factors and amphioxus density and biomass were calculated using SPSS. Table 1 shows the correlation matrix of aquatic environmental factors, including water temperature (T), dissolved oxygen (DO), pH, salinity (S), nitrate nitrogen (NO_3_-N), nitrite nitrogen (NO_2_-N), ammonia nitrogen (NH_3_-N), phosphorus (PO_4_-P), and amphioxus density and biomass (Table 1).

**Table 1:** Correlation matrix of aquatic environmental factors.

	*T*	pH	S	DO	PO_4_-P	NO_2_-N	NO_3_-N	NH_3_-N	Density	Biomass
T	1	−0.293	−0.768**	0.090	0.110	−0.019	0.226	−0.162	−0.525	−0.519
pH		1	0.008	0.282	−0.297	−0.239	−0.237	−0.187	0.403	0.480
S			1	−0.252	0.204	0.216	−0.088	0.346	0.460	0.395
DO				1	−0.184	−0.240	−0.224	0.003	−0.386	−0.305
PO_4_-P					1	0.503*	0.502*	0.690**	−0.247	−0.322
NO_2_-N						1	−0.084	0.724**	−0.113	−0.191
NO_3_-N							1	−0.192	0.164	0.131
NH_3_-N								1	−0.394	−0.462
Density									1	0.992**
Biomass										1

* Two-tailed test *P *< 0.05.

** Two-tailed test *P *< 0.01.

The results did not show a significant correlation between aquatic environmental factors and amphioxus density or biomass. However, water temperature and salinity showed a significant negative correlation (*P *<* *0.01), as did active phosphate and nitrate nitrogen, nitrite nitrogen (*P *<* *0.05) and active phosphate, nitrite nitrogen and ammonia nitrogen (*P *<* *0.01).

#### Relationship between sulfide of sediment and amphioxus distribution

**Figure 4:**
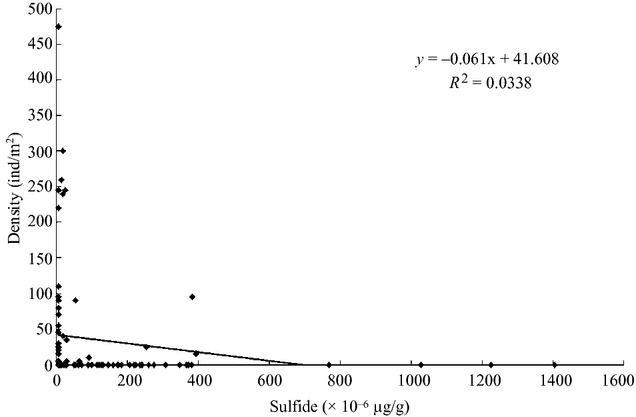
Hierarchical clustering of average density and biomass of Branchiostoma belcheni and sand samples (The figure shows the results of Hierarchical clustering and indicates that the distribution of *B. belcheri tsingtauens* was most closely related to sand percentage in granularity of 0.063–0.5 mm).

There was no significant negative correlation between the distribution of amphioxus species and the sulfide content in sediment (*R*^2^ = 0.0338, *P *>* *0.05; Fig. 4).

The level of the sulfide content is an important index for evaluating the quality of the marine sediment environment. The sulfide content was negatively correlated with benthic biomass and showed a large impact on oxygen consumption rate. Xiao (Lanfang 1998) showed that when the sulfide content reaches 400 × 10^−6^ μg/g – 1500 × 10^−6^ μg/g, the oxygen consumption rate is at its maximum level. When the sulfide content is higher than 1700 × 10^−6 ^μg/g, the biomass is <1 g/m^2^ and the sedimentary environment is essentially abiotic. In recent years, the sulfide content in the sediments in the Changli Sea area has met the first grade marine sediment quality standard. In addition, there was no significant negative correlation between the sulfide content and amphioxus density. We therefore conclude that the sulfide content is not a significant factor in the amphioxus population decline.

#### Relationship between grain size of sediment and amphioxus distribution

**Figure 5:**
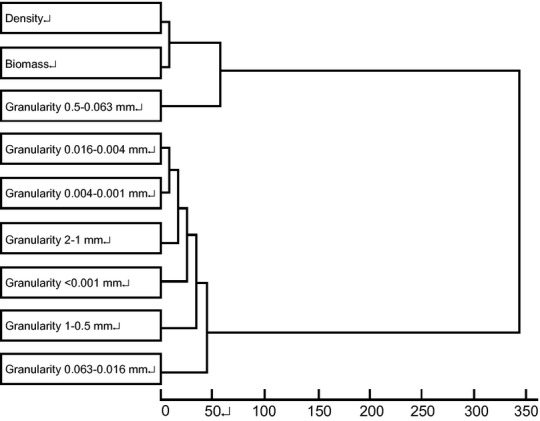
Hierarchical clustering of average density and biomass of Branchiostoma belcheri and sand samples (The figure shows the results of hierarchical clustering and indicates that the distribution of *Branchiostoma belcheri tsingtauense* was most closely correlated to the percentage of sediment with granularity 0.063–0.5 mm).

Sand samples were dried and filtered through sieves of decreasing mesh size. The percentages of different grain sizes were calculated and compared with the average biomass and resource density of amphioxus. Hierarchical clustering of these data showed that the distribution of *B. belcheri tsingtauense* was most closely correlated with sand grains between 0.063 and 0.5 mm, and the correlation was significant (Fig. 5).

**Figure 6:**
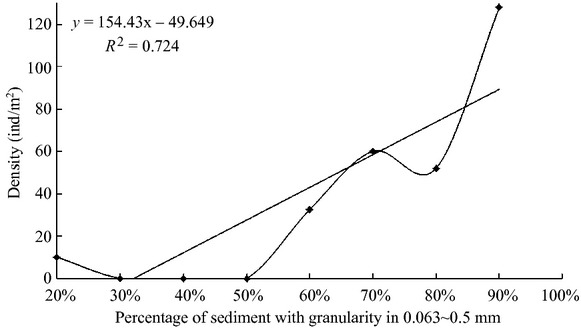
Numerical relationship between amphioxus density (ind/m^2^) and the percentage of sediment with granularity 0.063–0.5 mm (The figure shows the correlation between population distribution of amphioxus and variation in sediment types).

Relativity analysis of amphioxus density and the percentage of sediment with granularity 0.063–0.5 mm showed a significant positive correlation (*P *<* *0.01). When the percentage of sediment with granularity 0.063–0.5 mm was <50%, amphioxus density was <10 ind/m^2^; when the percentage of sediment with granularity 0.063–0.5 mm was >50%, amphioxus density was >60 ind/m^2^; and when the percentage of sediment with granularity 0.063–0.5 mm was >90%, amphioxus density exceeded 120 ind/m^2^ (Fig. 6).

### Spatial distribution of sediment

Three particle types comprise the sediment in this region, namely sand (S), silty sand (TS), and clayey sand powder (YT), and they comprise 80%, 15%, and 5% of the sediment, respectively.

Sediment particle size ranges from 0.001 to 1 mm, and the primary particle size is 0.063–0.5 mm. The lithology is primarily composed of fine sand and midsand, and *B. belcheri tsingtauense* is most densely distributed in areas where >90% of the sediment is comprised of this primary particle size.

**Figure 7:**
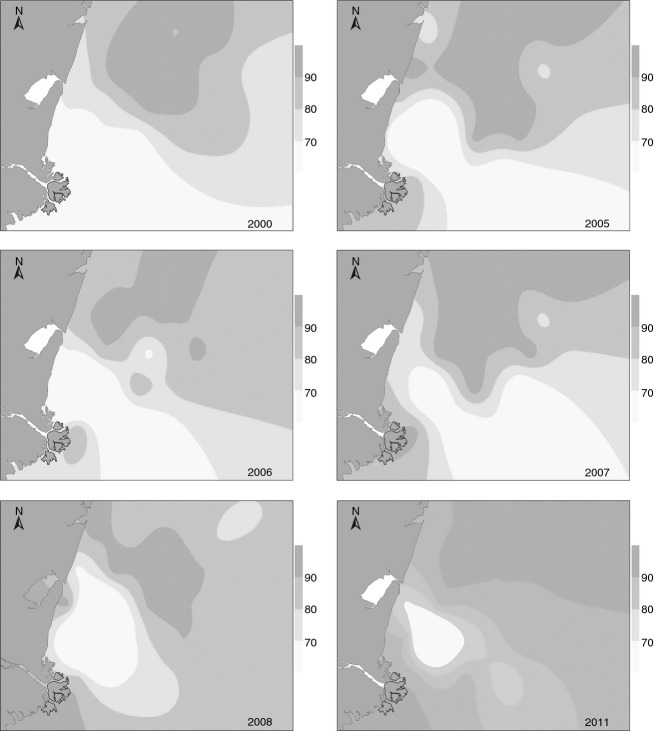
Distribution trends in primary component of sediment (particle size is 0.063–0.5 mm) in August from 2000 to 2011 (The varying shades of gray indicate differing percentages of the ideal sand particle size, 0.063–0.5 mm).

In 2000, the primary particle size made up more than 80% of the sediment in most of the monitoring area and more than 90% of the sediment where the seabed was 5–10 m deep. The main component was decreasing from 2005 to 2007; distribution area was further shrinking and fragmented, and moved to Dapu River Estuary integrally. As the favored sediment type shifted to the Dapu River Estuary, the amphioxus population also shifted in 2011 (Fig. 7).

## Discussion

### Features of regional distribution

Amphioxus are primarily distributed in areas where the seabed is 5–10 m deep, between the Xinkai Estuary and the Dapu River Estuary, where the sediment is primarily composed of medium and fine sand with particle sizes ranging from 0.001 to 1 mm. The highest density distribution of amphioxus occurs where >90% of the sediment particles are between 0.063 and 0.5 mm. Amphioxus density is significantly correlated with the sediment particle size. The density and biomass of *B. belcheri tsingtauense* have shown a recent, rapid decline in the Luan River Estuary.

### Impacts on distribution and survival of amphioxus

Our survey of the amphioxus population status and its environment showed that changes in the sedimentary environment may affect the amphioxus population distribution. Webb suggested that sediment type is important in determining the benthic distribution of amphioxus (Webb 1975). The results of hierarchical clustering and relativity analysis revealed a significant positive correlation between amphioxus density and primary particle size of the sediment, and that changes in the grain size composition of sediment may significantly influence the population distribution and density of amphioxus. In recent years, the amphioxus habitat has changed drastically (Fig. 7); the changes to the sediment in this region primarily result from the reduction in sediment discharge from Luan River and expansion of the raft-cultivation area.

#### Reduction in sediment transportation

Luan River is the major sand input source for the amphioxus habitat. It is the largest river in northern Bohai Bay, with an annual flow averaging 45 × 10^8^ m^3^ and an annual sediment load of 19 × 10^6^ t, which accounts for 98% of the total discharge into the sea (Jinliang and Wen 1997).

With recent reservoir construction (Panjiakou, Daheiting and Taolinkou) and the operation of the Luan River–Tianjin Water Diversion Project in the upper reaches of Luan River, estuarine water flow of the river has been sharply reduced by cross-regional water transfer and river closure.

Although total water flow of Luan River still exceeds 70% of its previous volume, water flow into the sea is now <30%. Water flow of Luan River into the sea was 7.96 × 10^8^ m^3^ in the 1980s (53.42 × 10^8^ m^3^ in the 1950s, 37.9 × 10^8^ m^3^ in the 1970s) (Haining 1999). The years between 1994 and 1996 were wet, and water flow into the sea was 22.5 × 10^8^ m^3^ in 1990s, approaching the values seen in the 1980s but still below the average level of the 1950s. In drought years, runoff into the sea is close to zero. Before 2002, the average annual runoff of Luan River was 21.28 × 10^8^ m^3^ (as measured at the hydrological stations of Luan county from 1980 to 2002) (Xianhan et al. 1999). Overall, average water flow of Luan River into the sea has declined each year. In addition, from 1980 to 2000, reservoirs upstream in Luan River were required to use dead storage to supply cities each year, which also indicates that water input was declining. Aiwen and Guobiao (Aiwen and Guobiao 1997) have predicted that the temperature of the Luan River area will rise by 1.2°C in coming years, that annual precipitation will increase by 1%, and that because evaporation exceeds precipitation, annual runoff will decline by 2.53 × 10^8^ m^3^ (a reduction of 46%) in the Luan River basin. As flood control projects and water storage lakes were constructed upstream, bank erosion declined and average sediment concentration decreased (Xiaolan and Dongda 2006). The average sediment load into the sea also decreased from 24 × 10^6^ (1950s) to 0.41 × 10^6^ t (1980s) (Yixu et al. 1994).

Because of the decline in terrestrial sediment supply, the region where >90% of the sand particles were 0.063–0.5 mm shifted to the northern Dapu River Estuary. The area between the Xinkai Estuary and the Dapu River Estuary was larger than that of Xinkai Estuary coastal area. As the sediment load declined, the amount of sand deposited near shore was sharply down by tide and sandy beach siltation gradually ceased, breaking the cycle of sediment accumulation between the land and the sea. From 2005 to 2008, the area where the percentage of sediment with granularity 0.063–0.5 mm exceeded 90% lay 5–15 m deep outside the Xinkai Estuary, with no significant change from year to year. As the sediment load of Luan River altered greatly from 2000 to 2004, the size of the primary sediment particles also changed. There were many reasons for the amount of water and sediment had significantly reduced, in consequence surface runoff changed by human activities and global climate change. From our results, it appears that this reduction in the sediment load may influence amphioxus populations and its habitats.

#### Rapid development of mariculture

As the area of shellfish culture has expanded, it has strongly affected the sediment environment. In areas of dense shellfish aquaculture, the biological settlement effect is very apparent (Rubao et al. 1998). Joint action of tides, ocean currents, etc., resuspend sediment particles in the water, resulting in a high concentration of suspended particles. As scallops do not filter all suspended particles, a portion of the suspended particles become false dung (biogenic sediment) (Thomas et al., 1995). The higher concentration of suspended particles in the water, the higher proportion of false dung. In addition, a portion of the filtered particulate also became feces, which were not absorbed. The process of false dung and feces settlement is called biological settlement (Minghua et al. 2004). The subsidence rate of these smaller particles is more rapid than that of the original suspended particles, resulting in stabilization of the rheological properties. Settlement in seawater culture zones is much greater than that in nonculture zones. For example, Hatcher investigated Upper South Cove mussel culture zones in Canada and found that settlement in culture zones is often greater than that in nonculture zones (Hatcher et al. 1994). Peibing and Jiwu (2001) showed that biological settlement caused by shellfish culture not only increased the quantity of seabed sediment but also altered the soft coral sediment components. Kaspar et al. (1985) observed that sediment from shellfish aquaculture changed sediment characteristics in Sweden; after each breeding season, the sediment in mussel culture zones thickened by approximately 10 cm. In my opinion: Excessive levels of microbial decomposition increased feces and decaying matter in sediment, thus increasing the component of small particles and clay. Thus, in scallop culture zones, the small-particle component of sediment increases.

**Figure 8:**
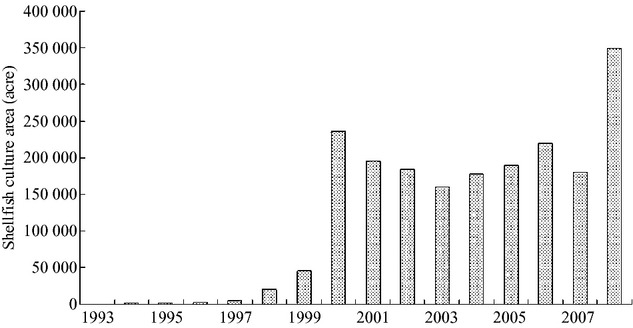
Shellfish raft culture area in Changli from 1993 to 2010 (The histogram indicates variation in annual area used for scallop culture in the Luan River Estuary).

In recent years, with the rapid development of aquaculture, Changli County (which has jurisdiction over the Luan River Estuary) has become the epicenter of the mariculture industry in Hebei Province. Shellfish culture began in 1993, and the period of rapid development commenced in 1999, with the area under culture increasing sharply to 7 661 acres (Lianzhen 2006). During this period, the Changli County government proposed a new target to “Construct a Marine County” and develop aquaculture and fishing. The area under culture reached 39,000 acres in 2000. From 2000 to 2010, the area of floating raft cultivation was maintained at a high level, with a peak of 107,000 acres in 2010. This rapid development continues (Fig. 8).

From 2000 to 2007, as shellfish agriculture expanded, the original unified and symmetrical area most favorable for amphioxus growth became fragmented (Fig. 7). The phenomenon was most obvious in 2006. *B. belcheri tsingtauens* was previously centered outside the Xinkai Estuary in the area 5–15 m deep, but became rare in 2006; sediment analysis showed that the 0.063- to 0.5-mm-diameter sediment component was 66.29%, and the content of silty sand had increased. Previous studies have shown that this type of sediment environment is unsuitable for amphioxus. Their results were similar to that of Hatcher's: Changes in sediment character directly affect sediment components and distribution, and the concentration of fine particles is generally higher in shellfish culture zones (Hatcher et al. 1994). In addition, installation of large quantities of breeding rafts and culture cages have altered the hydrodynamic conditions as well as sand transport and metastasis, which further discourage the formation of favorable amphioxus habitat. Since 2003, as sediment types have changed, the spatial distribution of the amphioxus population has diffused from a core area outside the Xinkai Estuary at a depth of 5–15 m to neighboring areas due to the increase in scallop culture rafts.

**Figure 9:**
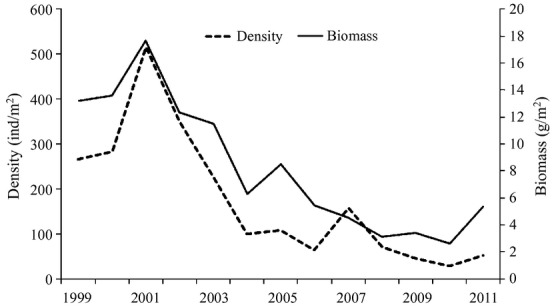
Trends in amphioxus density and biomass in August from 1999 to 2011 (The density and biomass of amphioxus changed annually from 1999 to 2011; dotted lines indicate density and solid lines represent biomass).

We also observed a lag between the development of aquaculture and its influence on amphioxus. From 1999 to 2001, amphioxus density and biomass showed an ascendant trend, during which time the scallop farming area had just begun to grow, and the environmental adaptation of active and passive migration had a certain hysteresis, indicating that the impact was not immediate. With passing years, the impact became evident in 2003. Scallop farming reached a peak in 2008, at the same time that amphioxus density and biomass reached their lowest point (Figs 7 and 9).

In conclusion, our results indicate that both sediment discharge in the upper reaches of Luan River and rapid expansion of the scale of raft-breeding mariculture are the primary factors that destroy the amphioxus habitat. However, further investigation is needed.

## Supporting Information

**Figure S1.** Changes in the sedimentary environment may affect the amphioxus population distribution.
