# Supplementary figures and images for: Temporal and spatial dynamics of amphioxus population (Branchiostoma belcheri tsingtaneuse) and its influential factors in Luan River Estuary, China
Source: Ecol Evol. 2014 Jul 7;4(15):3027–37. doi: 10.1002/ece3.1152 (PMC4161176; doi:10.1002/ece3.1152)

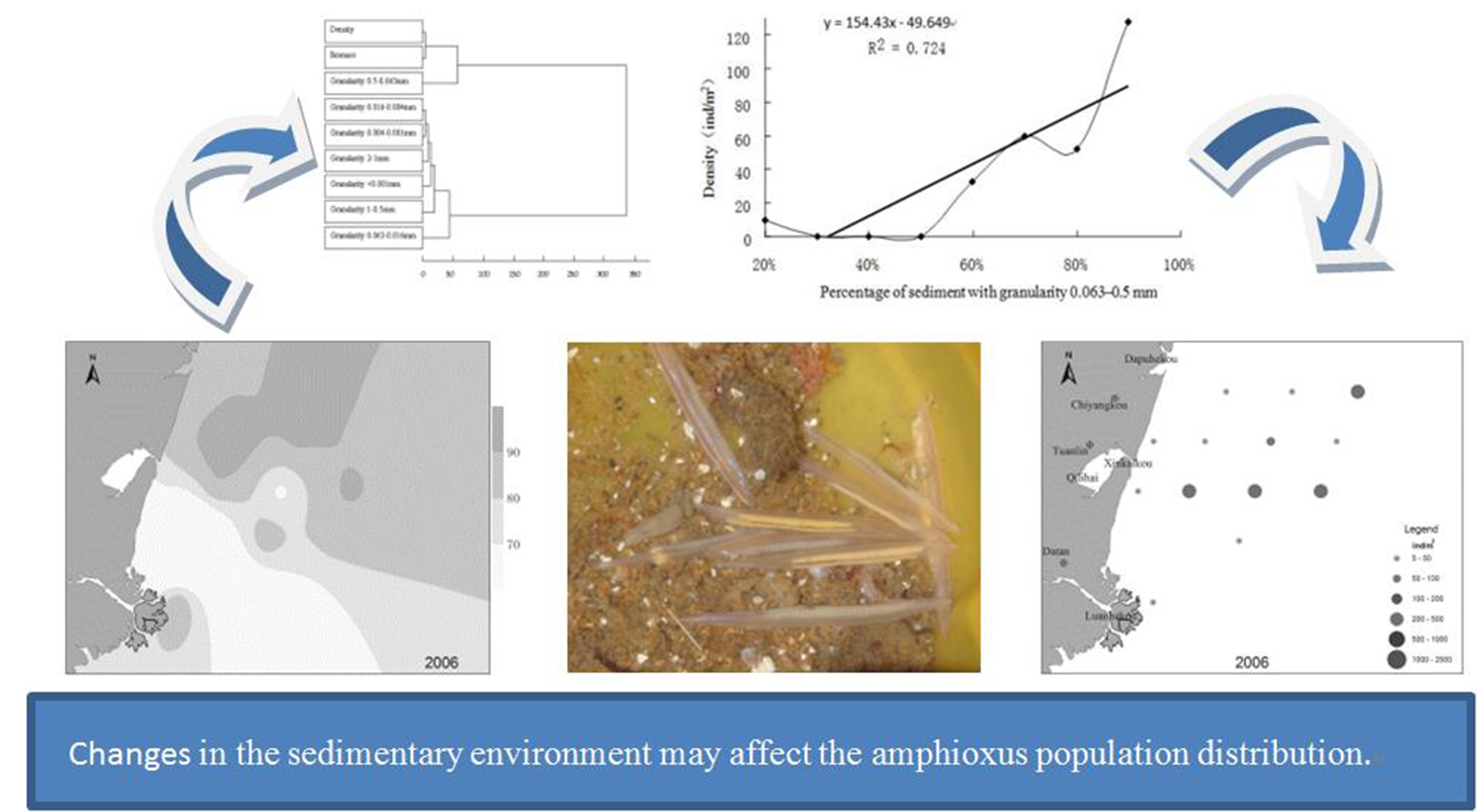

Supplement: Supplementary file 1 — Figure S1. Changes in the sedimentary environment may affect the amphioxus population distribution. [file ece30004-3027-sd1.tif]
